# Pressure Engineering Promising Transparent Oxides with Large Conductivity Enhancement and Strong Thermal Stability

**DOI:** 10.1002/advs.202202973

**Published:** 2022-09-30

**Authors:** Xuqiang Liu, Mingtao Li, Qian Zhang, Yiming Wang, Nana Li, Shang Peng, Tao Yin, Songhao Guo, Ye Liu, Limin Yan, Dongzhou Zhang, Jaeyong Kim, Gang Liu, Yandong Wang, Wenge Yang

**Affiliations:** ^1^ Key Laboratory for Anisotropy and Texture of Materials Northeastern University Shenyang 110819 China; ^2^ Center for High Pressure Science and Technology Advanced Research Shanghai 201203 China; ^3^ School of Materials and Chemical Engineering Zhongyuan University of Technology Zhengzhou 451191 China; ^4^ Hawaii Institute of Geophysics & Planetology University of Hawaii Manoa Honolulu HI 96822 USA; ^5^ Department of Physics and Institute for High Pressure Hanyang University Seoul 04763 South Korea

**Keywords:** electrical conductivity, high pressure, optical transmission, thermal stability, transparent conducting oxides

## Abstract

Transparent conducting oxides (TCO) with high electrical conductivity and high visible light transparency are desired for a wide range of high‐impact engineering. Yet, usually, a compromise must be made between conductivity and transparency, limiting the practical application of a TCO to the next level. Furthermore, TCO performance is highly sensitive to composition, so conventional synthesis methods, such as chemical doping, cannot unravel the mysteries of the quantitative structure–performance relationship. Thus, improving the fundamental understanding or creating materials‐by‐design has limited success. Here, a strategy is proposed to modulate the lattice and electronic and optical properties precisely by applying pressure on a TCO. Strikingly, after compression–decompression treatment on the indium titanium oxides (ITiO), a highly transparent and metastable phase with two orders of magnitude enhancement in conductivity is synthesized from an irreversible phase transition. Moreover, this phase possesses previously unattainable filter efficiency on hazardous blue light up to 600 °C, providing potential for healthcare‐related applications with strong thermal stability up to 200 °C. These results demonstrate that pressure engineering is a clean and effective tool for tailoring functional materials that are not achievable by other means, providing an exciting alternative property‐tuning dimension in materials science.

## Graphical abstract

A metastable state in transparent conductive oxide is achieved by a precise pressure engineering design, which can efficiently block the blue light. After the compression–decompression cycle treatment, the material shows high optical performance with a two orders of magnitude enhancement in conductivity. The high‐pressure‐synthesized phase also exhibits strong thermal stability up to 200 °C, promising for future multifunctional high‐temperature applications.

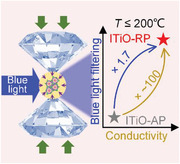

## 1 Introduction

Advances in transparent conductive oxide (TCO) have allowed the rapid development of a wide range of technologies, including low‐e windows, transparent contacts for solar cells, optoelectronic devices, organic light‐emitting devices, organic photovoltaics, and transparent field‐effect transistor.^[^ ^1^ ^–^ ^4^ ^]^ These oxides have been studied for nearly a century, and currently, the overall transparent electrode market is worth over US$5 billion per year.^[^ ^5^ ^]^ To further approach the performance limit of transparent electrodes, we must synergistically improve TCO electrical conductivity and optimize visible‐light transparency.^[^ ^6^, ^7^, ^8^ ^]^

However, high conductivity and transparency are generally exclusive due to electronic structure regulation.^[^ ^9^ ^]^ According to the Drude model, the conductivity is positively related to the carrier concentration and mobility.^[^ ^10^ ^]^ Thus, many free carriers (electrons) lead to strong light absorption, so the carrier concentration inevitably limits optical transparency.^[^ ^11^ ^]^ Therefore, increasing the conductivity without sacrificing the transparency can only be obtained by increasing the carrier mobility to be as high as possible. In this context, various complicated compositional modifications, including different‐elements‐doped oxides or combinations of oxides, have been investigated.^[^ ^12^, ^13^, ^14^, ^15^ ^]^ Yet, these strategies inevitably face the challenge that although the theoretical maximum carrier concentration is extremely high in terms of dopant solubility, the carrier mobility in TCO is further limited by the ionized impurity scattering process^7^, deteriorating the required electronic properties. Overcoming the compromise between high conductivity and high transparency in TCOs remains challenging due to the lack of complete structure‐property relationship data, even for well‐studied indium‐based oxides, which is a critical hindrance for in‐depth materials‐by‐design studies. Re‐scrutinizing the structures of materials is an opportunity. For example, indium‐based oxides usually crystallize in the cubic bixbyite‐type structures with a space group of *Ia* $\bar{3}$ at ambient conditions.^[^ ^16^, ^17^ ^]^ Such a ground phase has been well investigated, while other possible metastable states of this TCO have been severely overlooked. Considering that the polymorphism of the crystal structure may provide diverse physical properties, these metastable materials could have excellent properties that are absent in thermodynamically stable phases.^[^ ^18^, ^19^, ^20^, ^21^ ^]^ Therefore, there is a chance to tune the structure and thus, achieve novel properties in known TCOs via their possible metastable states.

Recent reports have suggested that several high‐energy light‐induced degradation effects in solar cells may be detrimental to maintaining long‐term device efficiency.^[^ ^22^, ^23^, ^24^ ^]^ Blue light, as a high‐energy visible light with a wavelength of 400–500 nm, should be paid attention to when evaluating the transparency of transparent electrodes. In addition, blue light could regulate human energy and rhythm during daytime,^[^ ^25^ ^]^ but excessive exposure to hazardous blue light (400–460 nm) may cause potential damage to photoreceptor cells in the retina.^[^ ^26^, ^27^, ^28^ ^]^

Pressure is a powerful and clean tool for tuning the crystal lattice substantially, enabling unprecedented property variation as strong functions of compression.^[^ ^29^, ^30^, ^31^ ^]^ In recent years, pressure engineering has demonstrated a promising path to pinpoint the properties of functional materials with atomic‐level understanding.^[^ ^32^, ^33^, ^34^ ^]^ However, pressure‐driven structure transformation and phase transition are usually reversible. Thus, employing pressure as an effective post‐synthesis method must ensure that the high‐pressure metastable states can be retainable to ambient pressure in their application temperature range.^[^ ^35^ ^]^

In this work, we discovered a pressure‐driven irreversible phase transition in classic TCO material, indium titanium oxides (ITiO), which trap materials in their metastable phases with novel properties inaccessible via other routes. We present a first example of large conductivity enhancement by up to two orders of magnitude from stable to metastable states (denoted as ITiO‐RP), which is physically different from any previous results realized via element doping. More exceptionally, the metastable ITiO‐RP phase exhibits nearly no loss in average transmittance compared to as‐prepared ITiO (ITiO‐AP), significantly blocking hazardous blue light (>460 nm) and demonstrating strong thermal stability over the temperature range of 25–200 °C. The density functional theory (DFT) calculations show that in Ti‐ and Sn‐doped In_2_O_3_, the effective mass of electrons in the metastable phase is lighter, theoretically supporting for the enhanced conductivity by pressure engineering. These results provide an alternative route to advance transparent electrode technology significantly.

## 2 Results and Discussions

### 2.1 Pressure‐Driven Functionality Enhancement

**Figure 1:**
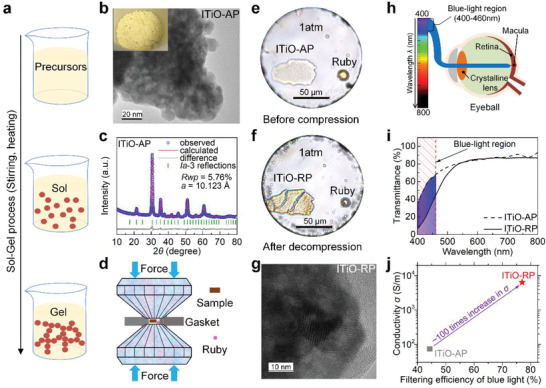
Sample preparation, characterization, and performance for as‐prepared (AP) and high‐pressure released (RP) indium titanium oxide (ITiO) samples. a) Schematics illustrating the sol–gel synthesis of the ITiO nanocrystals. b) Transmission electron microscopy image of the ITiO‐AP at room temperature. Upper‐left corner displays the yielded ITiO‐AP product with a light yellow color. c) Rietveld refinement results for ITiO‐AP. The R‐weighted pattern (Rwp) was calculated to evaluate the precision of the model compared to observed data. d) Schematic of generating high‐pressure conditions in the sample chamber by compressing the diamond anvil cell (DAC). e,f) The pictures of the ITiO‐AP and ITiO‐RP in the sample chamber. g) Transmission electron microscopy image of the ITiO‐RP at room temperature. h) Schematic illustrations for the potential blue‐light hazard to human eyes. Exposure to blue‐light hazard regions may cause phototoxic retinal damage. i) Comparison of the ITiO‐AP and ITiO‐RP transmittance. The average transmittance data are shown in Table 1. j) Comparison of the filtering efficiency in the blue‐light hazard region and electrical conductivity between ITiO‐AP and ITiO‐RP.

We employed the sol–gel method to synthesize a representative TCO, indium titanium oxide nanocrystals (**Figure** **1**a, see the Experimental Section for details). Figure 1b and Figure S1a (Supporting Information) show the transmission electron microscopy (TEM) images of the ITiO‐AP, from which the particle size is estimated to be 10–20 nm. We performed X‐ray diffraction (XRD) to examine the purity and crystal structure of the ITIO‐AP. As shown in Figure 1c, the results showed a good fit between the observed pattern and calculated result using a cubic *Ia* $\bar{3}$ model, as evidenced by the sufficiently small discrepancy factors: R‐Weighted Pattern (Rwp) = 5.76%. The crystal lattice parameter was resolved to be *a* = 10.123 Å, similar to the report on cubic In_2_O_3_.^[^ ^16^ ^]^ As seen in Figure S1b, the observation of diffraction patterns of (211), (222), (400), and (433) from the cubic phase by the selected area electron diffraction (SAED) further verifies the crystal structure of the cubic ITiO phase. High‐pressure diamond anvil cell experiments were conducted up to 55 GPa (Figure 1d), after which the applied pressure was totally removed. As shown in Figure 1e,f, the color of the ITiO‐AP sample changes from light to dark yellow after pressure treatment, indicating more absorption in the blue‐light range and thus, implying its potentially irreversible optical properties. Note that a small ruby ball was placed next to the sample for pressure calibration. Figure 1g shows the TEM image of the ITiO‐RP sample. The decompressed material consists of nanoparticles with very small particle size, as in the case of the starting material (Figure 1b and Figure S1a, Supporting Information). This indicates that the nanocrystals of ITiO were not simply merged after pressure treatment.

Blue light has the highest photon energy in the visible spectrum and easily penetrates the cornea and lens to reach the retina, potentially causing retinal damage, a phenomenon called “blue‐light hazard” by ophthalmologists (Figure 1h).^[^ ^26^ ^]^ Potential phototoxic retinal damage is expected to occur in the blue light spectrum at 400–460 nm.^[^ ^28^ ^]^ Figure 1i shows the transmission spectra of ITiO‐AP and ITiO‐RP. We note that the attenuation of transparency is mainly concentrated in the wavelength range of blue‐light hazard. Here, the average transmittance $\tilde{T}$(%) can be calculated by

(1) $$\tilde{T}=\frac{\int_{\lambda_{1}}^{\lambda_{2}}T\left(\lambda \right)d\lambda }{\lambda_{2}-\lambda_{1}}$$

where *T*(*λ*) is the transmittance value for a certain wavelength (*λ*), and *λ*_1_ and *λ*_2_ are the minimum and maximum wavelength, respectively.^[^ ^36^ ^]^ As listed and highlighted in **Table** **1**, the $\tilde{T}$ of the blue‐light hazard region is greatly reduced from 55.63% for ITiO‐AP to 22.98% for ITiO‐RP. Therefore, ITiO‐RP's blue light filtering performance (77.02%) is superior to pristine ITiO‐AP. Importantly, this benefit does not sacrifice much of the optical transparency in other visible light wavelengths for ITiO‐RP, which retains as much as 81.31% optical transparency in the wavelength range of 460–800 nm. Moreover, both samples have nearly identical low work function value ≈4.3 V (see Figure S2 and related discussion for more details). Together with the lager conductivity enhancement (Figure 1j), these results prove pressure treatment can manipulate and improve TCO material, though more detailed investigations on pressure‐driven structural and functional evolutions are vital.

**Table 1:** Average transmittance of ITiO nanocrystals before and after pressure treatment

Samples	${\tilde{T}}_{\mathrm{Blue}-\mathrm{light}\;\mathrm{hazard}}$ (400–460 nm)	${\tilde{T}}_{\mathrm{Vis}}$ (460–800 nm)
ITiO‐AP	55.63%	84.36%
ITiO‐RP	22.98%	81.31%

### 2.2 Irreversible Pressure‐Driven Phase Transition

**Figure 2:**
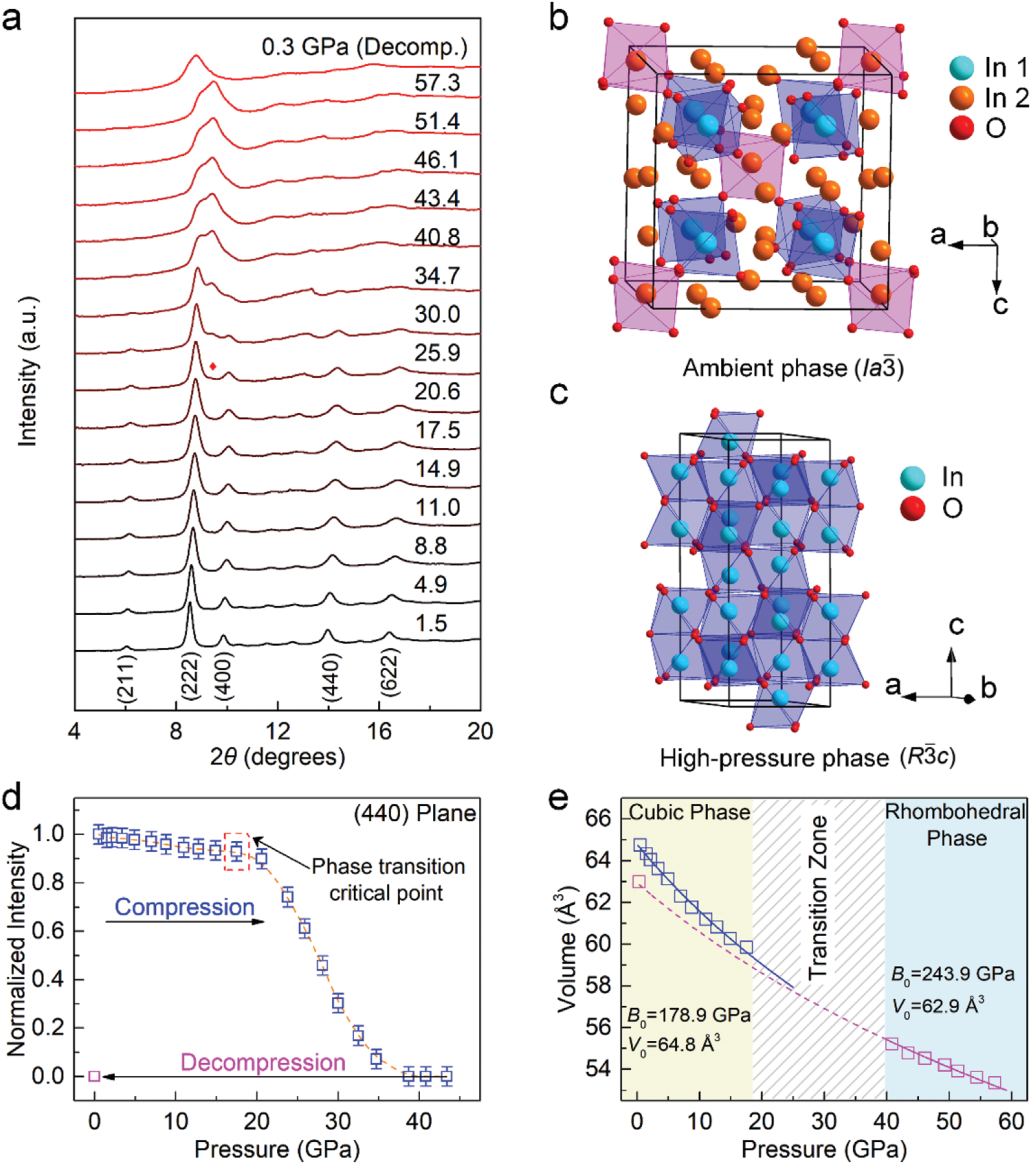
Pressure‐driven crystal structure evolution of ITiO. a) Synchrotron XRD patterns at various pressures. The peak with a red mark is the (110) plane of the *R* $\bar{3}$ *c* phase. b,c) The *Ia* $\bar{3}$ and *R* $\bar{3}$ *c* structures of ITiO. d) Normalized integrated (440) peak intensity of cubic phase as a function of pressure. e) Volume versus pressure of ITiO. Lines show the fitting results by the Birch‐Murnaghan equation of state.

To explore the origin of the functionality enhancement discovered in pressure‐engineered indium titanium oxides, we monitored the crystalline structure changes by in situ synchrotron XRD measurements. **Figure** **2**a shows selected synchrotron XRD patterns of an indium titanium oxide sample under a completed compression–decompression cycle with a peak pressure of 57.3 GPa. Below 17.5 GPa, the crystal structure can be readily resolved using the ambient cubic phase of pure indium oxide In_2_O_3_ with space group *Ia* $\bar{3}$, where two crystallographically nonequivalent indium atoms (both are 6‐coordinated) and only one type of 4‐coordinated oxygen atom form the A_2_B_6_ type octahedrons (Figure 2b). Note that relatively few titanium atoms only share A‐site positions with the host indium and thus, play a tiny role in the crystal structures. Instead, it is the fact that the titanium ions (0.74 Å) are smaller than the indium ions (0.94 Å) that allow the titanium atoms to substitute indium atoms in the In_2_O_3_ lattice without significantly affecting the lattice constant.^[^ ^15^ ^]^ As applied pressure further increases up to 25.9 GPa, an additional reflection (marked as red squares in Figure 2a) appears, suggesting a symmetry lowering. When Liu et al.^[^ ^37^ ^]^ and Qi et al.^[^ ^38^ ^]^ observed the pressure‐induced phase transition of In_2_O_3_ at 15–25 GPa, they found that the first distinct diffraction peaks of the high‐pressure *R* $\bar{3}$ *c* phase appeared on the right shoulder of the (222) peak of the cubic phase. Our refinement results (Figure S3) demonstrate a reconstruction‐type phase transition from a low‐pressure *Ia* $\bar{3}$ phase to a high‐pressure *R* $\bar{3}$ *c* phase, which is consistent with previous reports on the high‐pressure structure of pure indium oxides.^[^ ^37^, ^38^, ^39^ ^]^ The high‐pressure structure is isostructural with corundum‐Al_2_O_3_, which contains only trigonal biprism coordination In^3+^ (or Ti^4+^) ions within the lattice of O^2−^ ions, with O atoms located at Wyckoff positions 12c, and 18e (Figure 2c). Such a *Ia* $\bar{3}$→*R* $\bar{3}$ *c* phase transition straddles a wide pressure range from 25.9 to 43.4 GPa, as supported by the gradual disappearance of the (440) peaks of the *Ia* $\bar{3}$ phase (Figure 2d). The observed coexistence of the two phases over a wide range of pressures is attributed to deviatoric stress that is not uncommon in DAC experiments.^[^ ^40^, ^41^ ^]^ Finally, a single high‐pressure phase was obtained at the highest pressure achieved in our experiment, 57.3 GPa, confirming that the phase transition was complete. Fitting the *P*–*V* curve to the second‐order Birch‐Murnaghan equation of state (EOS)^[^ ^42^ ^]^ gives the bulk modulus *B*_0_ = 178.9 ± 5.4 GPa of the low‐pressure phase and 243.9 ± 6.9 GPa of the high‐pressure phases, implying that the high‐pressure phase is less compressible and, thus, has pressure‐driven hardening behavior (Figure 2e).

To elucidate the experimental observations in ITiO, we further performed DFT calculations. The relative enthalpies versus pressure of the *Ia* $\bar{3}$ and *R* $\bar{3}$ *c* phases are shown in Figure S4 (Supporting Information). The calculated results indeed confirm that ITiO undergoes the *Ia* $\bar{3}$→*R* $\bar{3}$ *c* phase transition at about 12 GPa, consistent with the XRD result. Note that the Ti substitution into the In2 sublattice is lower in enthalpy at 0–35 GPa. At ambient pressure, the enthalpy difference for Ti substitution into the In1 and In2 sublattices is 170 meV per unit cell, showing that it is thermodynamically more stable when Ti atoms replace the In2 atoms. Therefore, in the following calculations, the Ti atom only replaces the In2 atom unless otherwise stated.

Previous reports on the compression behavior of In_2_O_3_ materials (see related discussion in the Supporting Information) presented controversial results about high‐pressure phase transition.^[^ ^37^, ^38^, ^43^, ^44^, ^45^, ^46^ ^]^ Therefore, more in‐depth studies are required for resolving the conflicting mysteries from existing studies. For our sample, only the new peaks of the *R* $\bar{3}$ *c* phase were found upon releasing pressure to ambient conditions (Figure 2a and Figure S3, Supporting Information), demonstrating that the high‐pressure phase survives after completely removing the applied pressure. We found a good agreement between the theoretical and experimental lattice volume of the ITiO‐RP sample, derived from the extrapolation of the EOS of the high‐pressure phase and directly obtained from XRD data (Figure 2e), respectively. HRTEM and SAED were also employed to further confirm the presence of the *R* $\bar{3}$ *c* phase in the ITiO‐RP sample (Figure S5, Supporting Information). Furthermore, consistent *d*‐spacing values were obtained between the SAED and the synchrotron XRD (Table S1, Supporting Information). Together with the in situ high‐pressure Raman results (Figure S6, Supporting Information) these results further support the irreversibility of the phase transition.

### 2.3 Optical and Electrical Characterizations

If a crystal could be retained in the novel phase, the physical properties upon pressure‐induced structural reconstruction could reach previously unattainable magnitudes, at which the optical and electronic functionalities can reach their optimized level.

**Figure 3:**
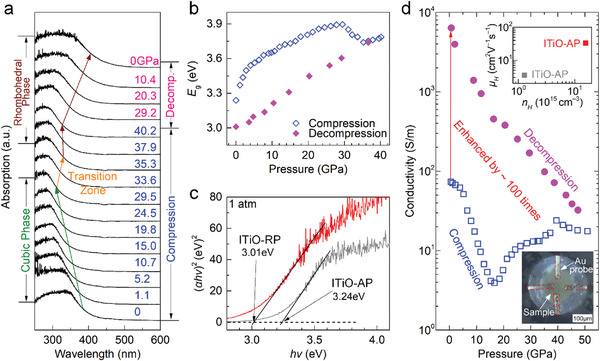
Variations of optical and electrical properties under high pressure for ITiO. a) Selected optical absorption spectrum at high pressures. b) Bandgap evolution under pressure. c) Determination of the bandgap for ITiO‐AP and ITiO‐RP. d) Conductivity of ITiO as a function of pressure during compression and decompression. The upper‐inset shows the hall mobility *µ*_H_ and carrier concentration *n*_H_ for ITiO‐AP and ITiO‐RP, both of which show a 10‐fold increase. The bottom‐inset shows a configuration of ITiO samples in the DAC sample chamber with four Au probes.

**Figure** **3**a and Figure S7 (Supporting Information) show the optical absorbance spectra and optical images of an ITiO sample under a compression–decompression cycle. We witnessed a pressure‐dependent behavior, which can be understood from the lattice structure's pressure changes that subsequently redefines the boundary conditions for the electronic wavefunctions. Then, the magnitudes of the direct bandgap *E*_g_ can be estimated by extrapolating the linear portion of the Tauc plots to the baseline. As summarized in Figure 3b, the bandgap of ITiO experienced a blueshift from 3.24 to 3.90 eV as hydrostatic pressure was increased from 1 atm to 29.5 GPa, followed by a redshift down to 3.74 eV at 35.3 GPa, and then increases slightly up to 3.79 eV over 35.3–40.2 GPa.

These optical evolutions are consistent with the pressure‐driven structural changes. We also employed DFT calculations to investigate the evolution of the band structure under pressure in the low‐pressure *Ia* $\bar{3}$ and high‐pressure *R* $\bar{3}$ *c* phases. The band structures of these two phases (Figure S8, Supporting Information) show the pronounced feature of a Moss–Burstein shift,^[^ ^47^ ^],^ i.e., the Fermi level shifts above the conduction band minimum (CBM) after doping, which is similar to the theoretical prediction of Sn‐ and Mo‐doped In_2_O_3_.^[^ ^48^, ^49^ ^]^ Furthermore, the two observed blueshift regimes under compression and decompression in the *Ia* $\bar{3}$ and *R* $\bar{3}$ *c* phases correspond well to the evolution of the theoretical optical bandgap of both phases tuned by external pressure, as shown in Figure S9 (Supporting Information). As pressure was applied, the valence band maximum (VBM) of the *Ia* $\bar{3}$ phase, which is mainly contributed by the electrons from the O atoms, moved down while its conduction band minimum (CBM) remained constant. This leads to the bandgap widening, which agrees with the blueshift trend from our optical absorbance study. Note that the calculated optical bandgap of the *Ia* $\bar{3}$ phase is lower than that of the *R* $\bar{3}$ *c* phase from 0 to 30 GPa, which is the opposite of our experimental results and may be related to the tendency of DFT calculations to underestimate the bandgap.

The continuously decreasing bandgap further convinced the bandgap evolution's structural root upon decompression from the retainable high‐pressure phase (Figure 3a). While pressure is released, the shift of the VBM to high‐energy could reduce the bandgap. The electronic structure also exhibits retainable behavior like the crystal structure, as evidenced by the absorbance edge shifting to a smaller energy range after the pressure is released (Figure 3c), corresponding to a significantly smaller bandgap of 3.01 eV observed in the pressure‐engineered ITiO‐RP sample.

Electrical conductivity is an essential characteristic of transparent electrodes for almost all related applications. We carried out in situ resistance measurement using four‐point‐probe methods within a DAC device. Figure 3d shows the electrical conductivity change of an indium titanium oxide sample as a function of pressure upon compression and decompression. In the first pressure range over 1 atm to 16.7 GPa, the sample's conductivity gradually decreases from 73 to 4.9 S/m. As the pressure increases further up to 40 GPa, a pressure‐driven conductivity enhancement was found, followed by a slight drop from 40 to 51 GPa, where the pressure‐driven phase transition completes (Figure 2).

Considering the irreversible behavior of the pressure‐driven phase transition and the compression‐induced conductivity drop in the high‐pressure phase, we employed decompression to investigate the possible conductivity enhancement. As the applied pressure is gradually removed, the sample shows a rapid increase of conductivity from 18 S/m at 51 GPa to 6.5 × 10^3^ S/m at ambient conditions, achieving a surprising two orders of magnitude enhancement. Drude theory points out that the conductivity *σ* is determined by the carrier mobility *µ* and concentration *n* as *σ* = *neµ*, where *µ* and *n* can be derived from the electromagnetic characterization. We then employed the Hall effect and van der Pauw method to extract and compare these parameters between ITiO‐AP and ITiO‐RP. In ITiO‐RP both *µ* and *n* are ≈10 times higher than those of ITiO‐AP (Figure 3d), which are, thus, proposed to be the dominant contributors to the large conductivity enhancement. Based on the calculated band structures under pressure, the change of carrier mobility can be predicted by calculating the effective mass of the conduction band electrons. The electrons with low effective mass in CBM are not conducive to charge movement and thus, have low electron mobility. As shown in Figure S10, the pressure‐dependent effective masses (m*) along the three crystallographic directions are plotted. The results show that the calculated m* for the *Ia* $\bar{3}$ and *R* $\bar{3}$ *c* phases are almost isotropic. With the increasing pressure, the m* of both phases becomes heavier, which in turn reduces the carrier mobility *µ*. This may theoretically explain the observed decreasing *σ* trend of the *Ia* $\bar{3}$ and *R* $\bar{3}$ *c* phases upon compression by assuming that the carrier concentration *n* is almost constant. The predicted m* of the *R* $\bar{3}$ *c* phase is lighter than that of the *Ia* $\bar{3}$ phase at ambient pressure, which theoretically supports our conductivity measurements that the ITiO‐RP phase has higher conductivity.

Sn‐doped In_2_O_3_ (ITO) is the most widely used commercial material as transparent electrodes, in other words, ITO performance is equivalent to the ruler of transparent electrodes. Therefore, we compared the performance of ITiO‐RP and ITO to evaluate the effect of pressure engineering. The electrical conductivity and thickness of ITO film usually range from 5 × 10^−3^ to 1.25 × 10^6^ Ω^−1^ m^−1^ and from 2 to 200 nm, respectively.^[^ ^50^, ^51^ ^]^ For ITiO‐RP, its electrical conductivity is about 6.5 × 10^3^ Ω^−1^ m^−1^, which is quite rather similar to ITO. Since ITiO‐RP is a decompressed sample, its thickness is about 10 µm during resistance measurement. In addition, both ITiO‐RP and ITO exhibit up to 80% transmittance at 550 nm.^[^ ^51^ ^]^ Here, the optical transmittance (*T*) at 550 nm is selected as an uniform standard because it is close to the most sensitive wavelength of human eyes.^[^ ^8^, ^52^ ^]^ Note that in the characterization of optical properties, the thickness (*d*) of ITO is still 2–200 nm while the thickness of ITiO‐RP is about 5 µm. To quantitatively evaluate the performance of a transparent electrode material with different thickness, resistivity, and transparency, the figure of merit (*Φ*) proposed by Haccke is defined by

(2) $$\Phi =T^{10}/R_{\mathrm{sh}}=\sigma dT^{10}$$

where *R*_sh_ is the large sheet resistance.^[^ ^53^ ^]^ A higher *Φ* values indicate better performance of the material, which depends on two compromise properties of high transparency and low sheet resistance. The *Φ* of ITiO‐RP is estimated to 9.0 × 10^−3^ Ω^−1^, which is very close to the *Φ* values of spin coating ITO (11.9 × 10^−3^ Ω^−1^) and commonly sputtered but unannealed ITO layers (10.0 × 10^−3^ Ω^−1^).^[^ ^54^, ^55^ ^]^

**Figure 4:**
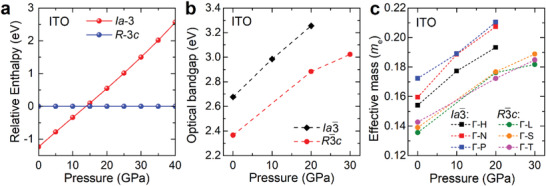
The theoretical results of ITO under pressure. a) Calculated enthalpies of *Ia* $\bar{3}$ phase (relative to *R* $\bar{3}$ *c* phase) for ITO as a function of pressure. b) The comparison between the calculated optical bandgap of *Ia* $\bar{3}$ and *R* $\bar{3}$ *c* phases at different pressures. c) Calculated effective mass for the conduction band electrons of two phases in ITO under pressure. Note that the effective masses along with the crystallographic direction [001], [011], and [111] correspond to the direction Γ→*H*, Γ→*N*, and Γ→*P*, for *Ia* $\bar{3}$ phase and Γ→*L*, Γ→*S*, and Γ→*T* for *R* $\bar{3}$ *c* phase, respectively.

To study the changes in the properties of ITO under pressure, we employed the DFT calculations to simulate the evolution the structural phase, the effective mass of electrons, and optical bandgap of ITO. The pressure‐dependent relative enthalpy of ITO (**Figure** **4**a) shows that above 14 GPa, *R* $\bar{3}$ *c* phase has the smaller enthalpy, theoretically predicting the pressure‐induce the *Ia* $\bar{3}$ → *R* $\bar{3}$ *c* phase transition. After analyzing the electronic structure in Figure S11, the optical bandgap and effective masses m* as a function of pressure are plotted in Figure 4b,c, respectively. Similar to ITiO, the m* and optical bandgap of the two phase in ITO increased with increasing pressure. At ambient pressure, the m* of *R* $\bar{3}$ *c* phase in ITO is lower than that of *Ia* $\bar{3}$ phase. Theoretically, this suggests that for ITO, the *R* $\bar{3}$ *c* phase has larger carrier mobility than *Ia* $\bar{3}$ phase, resulting in a higher electrical conductivity. This calculated result is consistent with other theoretical work on pure and doped In_2_O_3_.^[^ ^39^, ^56^, ^57^, ^58^ ^]^ Meanwhile, there is still a lack of experimental evidence that ITO with *R* $\bar{3}$ *c* phase is a TCO with better electrical and optical properties. To confirm whether our predicted optimization could be achieved by pressure engineering, it is calling for the follow‐up experimental characterizations on the crystal structure and physical properties of ITO under pressure.

### 2.4 High Thermal Stability in Pressure‐Engineered Oxides

**Figure 5:**
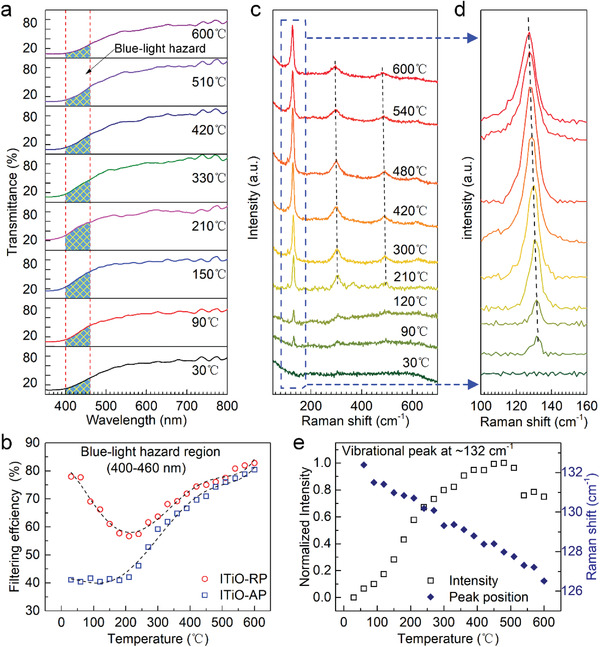
Thermal stability of optical applications for ITiO‐RP. a) Transmittance spectrum at selected temperatures. b) Blue‐light filtering efficiency as a function of temperature for ITiO‐AP and ITiO‐RP. The dashed lines are guide for eyes. c) Raman spectra of ITiO‐RP at different temperatures, and the magnified spectra in the range of 100–160 cm^−1^ are exhibited in (d). e) Normalized integrated vibrational peak intensity and peak position at ≈132 cm^−1^ as a function of temperature.

Thermal stability is an important property of TCOs affecting their applications, where low energy loss and less variation in properties as a function of temperature are highly desired. **Figure** **5**a and Figure S12 and S13 (Supporting Information) show the temperature dependence of the ITiO‐RP sample transmission, from which the blue‐light filtering efficiency was derived, stimulating us to investigate the thermal stability of the optical properties after pressure engineering. Figure 5b shows the temperature dependence of the blue‐light filter efficiency variations for ITiO‐RP and ITiO‐AP. We observed that for ITiO‐RP the filter efficiency decreases from 77% at room temperature to 55% at 200 °C, above which it monotonically increases up to 80% at 600 °C. Such a ≈25% variation is obviously less than that in ITiO‐AP (≈40%) over the same temperature range (Figure 5b and Figure S14, Supporting Information), demonstrating an improved, strong thermal stability.

We then conducted in situ high‐temperature Raman characterizations to explore the structural origin of the temperature‐dependent optical properties (Figure 5c,d). As temperature rises, there is a noticeable increase in the intensity of the Raman shift at about 130 cm^−1^ (Figure 5e). According to group theory, this low‐frequency Raman‐active *v*_1_ mode only exists in pristine *Ia* $\bar{3}$ Symmetry,^[^ ^59^, ^60^ ^]^ which indicates a temperature‐driven *R* $\bar{3}$ *c*→*Ia* $\bar{3}$ phase recovery in ITiO‐RP, leading to less ability to block blue light. To verify the kinetics of the phase transition, we collected the time‐dependent Raman spectrum of ITiO‐RP at the same invariable temperature at different times. The kinetics of this phase transition was checked at 60, 90, 120, 180, and 210 °C (Figure S15, Supporting Information). The results show that the temperature‐driven phase transition process may not be related to the heating time, supporting that the temperature is the key factor enabling this phase transition. We also monitored the change in the peak position of the *v*_1_ mode as a function of temperature. With the temperature rise, a redshift of the *v*_1_ Raman mode was observed, demonstrating a lattice‐expansion‐induced vibration softening, which is consistent with the previous report on the blueshift in indium oxides via pressure‐driven lattice‐shrinkage.^[^ ^38^ ^]^ The XRD results (Figure S16, Supporting Information) further support the proposed temperature‐driven phase transition and temperature‐dependent resistance (Figure S17, Supporting Information), proving that the sample transforms to the *Ia* $\bar{3}$ structure after compression–decompression followed by a heating‐cooling cycle. These results support that a metastable state can be trapped in the TCO material formed under pressure and survives over a temperature range of 25–200 °C, which is crucial for applications. Note that the term “metastable state” denotes the high‐pressure phase (ITiO‐RP), rather than the low‐pressure phase (ITiO‐AP). We also carried out a resistance measurement on this pressure‐temperature treated sample under pressure (Figure S18, Supporting Information). The result shows a more than 30‐fold improvement in conductivity after a cycle of compression and decompression, further confirming that conductivity enhancement originates from the crystalline phase change.

## 3 Concluding Remarks

By realizing an irreversible phase transition employing high pressure, we obtained metastable indium titanium oxides at ambient conditions with a conductivity enhancement of ≈100 times more than the pristine thermal stable phase, while maintaining their optical transparency with superior quality blue‐light blocking efficiency, and high thermal stability. The described high‐pressure room‐temperature process, or improvements thereof, could potentially convert TCO families into high‐performance composites suitable for electronic, optical, and energy applications, where healthcare use is especially important. Furthermore, our fabricated high‐performance materials could be scalable using the high‐temperature and high‐pressure process of large‐volume press high‐pressure facilities.^[^ ^61^, ^62^, ^63^, ^64^, ^65^ ^]^ Obtaining a large volume of pressurized TCO sample in this way could enable tunable, improved, and retainable performance.

Furthermore, we present efforts to understand the pressure‐driven stable‐metastable transition from a thermodynamic perspective, which offers a route to explore future high‐pressure synthesis in a broader context. We have shown that there should be a considerably high potential energy barrier between stable and metastable indium titanium oxides, as supported by the strong thermal stability in our pressure‐engineered samples. In addition, temperatures above 60 °C are sufficient to initiate the recovery of the stable phase. A key factor in achieving this reversible phase transition is temperature, which is kinetically low enough for sufficient atomic mobility and, thus, leads to structural instability at high temperatures. These results show that structural switching between the stable and metastable phase in ITiO can be realized using a compression and heating route. A similar phenomenon occurs in ZnO and CdTeMoO_6_,^[^ ^66^, ^67^ ^]^ which undergoes an irreversible pressure‐induced phase transition, and heating helps to recover it to the stable ambient phase. Therefore, a crucial guideline is derived for reaching a preserved metastable state with superior properties: it must have an obvious and tunable energy barrier between the metastable and ground state, depending on the detailed path of the structural transition. This concept could be extended to other functional materials beyond TCO materials.

## 4 Experimental Section

ITiO nanocrystals were synthesized by the sol–gel method. In detail, InNO_3_·4.5H_2_O ethanol solutions, which contain C_16_H_36_O_4_Ti with an atomic percentage Ti/(In+Ti) = 2.5 at%, were mixed with C_6_H_8_O_7_ ethanol solution at room temperature. Then, the mixed solutions were stirred at 323 K until gel formed. After that, the gel was dried at 393 K for 3 h to remove organic matter and water. The dried gel was further annealed at 773 K for 9 h before air cooling to room temperature. The phase purity of the as‐prepared ITiO nanocrystals was characterized by a laboratory Cu‐K*α* X‐ray diffractometer (XRD, PANalytical Empyrean) at ambient conditions. The morphology and composition were analyzed by scanning electron microscopy (SEM) with energy dispersion X‐ray spectrometry (EDS). The results are shown in Figure S19 and Table S2 (Supporting Information). The average Ti content is determined to be about 2.6 at%, consistent with the initial stoichiometric ratio.

Using a symmetric diamond anvil cell (DAC), in situ high‐pressure synchrotron X‐ray diffraction (XRD) measurements was carried at the 13‐BM‐C station of GSECARS, Advanced Photon Sources, Argonne National Laboratory. The culet of the diamond anvils had a diameter of 300 µm. A 250 µm thick rhenium gasket was used, which was first indented to about 40 µm thickness before laser‐drilling a 120 µm hole to serve as the sample chamber. Next, two diamond anvils with a culet size of 600 µm were used to pre‐press the powder for packing nanosized ITiO particles into a pellet with seamless contact and uniform thickness. The thickness of the compacted sample pellets was about 5 µm. Then, the sample pellets were loaded into the chamber with a small ruby ball attached to the culet. Neon was used as a pressure‐transmitting medium (PTM). The pressure was calibrated by the ruby fluorescence method.^[^ ^68^ ^]^

A monochromatic X‐ray beam with a wavelength of 0.4340 Å was employed in a pressure range of 0–60 GPa. The X‐ray detector was a MAR‐345 image plate. LaB_6_ powder diffraction was used as the standard to calibrate the sample‐detector distance using Dioptas software.^[^ ^69^ ^]^ The 2D diffraction images were also integrated using the Dioptas program.^[^ ^69^ ^]^ The lattice parameters were obtained by Rietveld refinements using the general structure analysis system (GSAS) package.^[^ ^70^ ^]^

High‐pressure electrical transport measurements were performed using the standard four‐probe method under van der Pauw configuration in a 2182A nanovoltmeter, a Keithley 6221 current source, and a 7001 switch system. The Hall effect measurements were performed in a commercial PPMS system (DynaCool, Quantum Design). A nonmagnetic BeCu DAC was used to generate high‐pressure conditions. A pair of 300 µm culet diamonds were used. A nonmagnetic BeCu thin shim with a thickness of 250 µm was used as a gasket. The pre‐indented hole was covered by cubic boron nitride (c‐BN) as an insulating layer (thickness ≈40 µm). Subsequently, a hole with a 150 µm diameter hole was laser drilled as the sample chamber, where the ITiO powder was fully loaded. A small ruby ball was put next to the powder sample for pressure calibration. No pressure transmitting medium was used in the high‐pressure conductivity measurements. Four thin gold wires with a diameter of 10 µm were used as the contact leads.

The Raman‐scattering spectra were collected using a Raman microscope spectrometer (Renishaw, U.K.) with backscattering geometry. A laser with a 532 nm wavelength was used as the excitation with 2400‐groves mm grating. For the high‐temperature Raman‐scattering measurement, a commercial Linkam stage was utilized with a precise temperature controller. The samples were heated up to 873 K. Each Raman scattering spectrum was collected after five minutes of temperature stabilization.

The transparency and absorbance measurements were conducted with a UV–vis absorption spectrophotometer with a light wavelength covering 250–860 nm. High‐temperature transmittance spectra were also collected using a UV–vis absorption spectrophotometer with a commercial Linkam stage. Each transmittance spectrum was collected after five minutes of temperature stabilization.

All calculations were done in the framework of density functional theory (DFT)^[^ ^71^, ^72^ ^]^ using the projector augmented wave (PAW)^[^ ^73^, ^74^ ^]^ pseudopotential method, as implemented in the VASP package.^[^ ^75^, ^76^ ^]^ The electron exchange‐correlation functional was treated in the generalized gradient approximation as proposed by Perdew–Burke–Ernzerhof (PBE). The valance electrons were considered as 4d^10^5s^2^5p^1^ for In, 3s^2^3p^6^4s^1^3d^3^ for Ti, 4d^10^5s^2^5p^2^ for Sn, and 2s^2^2p^4^ for O. The kinetic energy cutoff for the plane‐wave basis set expansion was set to 600 eV in all cases to avoid Pulay stress. A Γ‐centered Monkhorst‐Pack grid of 4 × 4 × 4 and 8 × 8 × 8 *k*‐points were used for the geometry optimization and calculation of DOS, respectively. The energy convergence of 1.0 × 10^−6^ eV was used for the electronic energy minimization steps. During relaxation, Feynman forces on each atom were minimized until they were less than 0.001 eV Å^−1^. To simulate the system with doping 2.6 at% Ti, a supercell was adopted with 16 formula units (containing 31 In atoms, 1 Ti atom, and 48 O atoms), and the actual Ti: (In + Ti) ratio was 1:32. To simulate the system with the best doping level of Sn (≈6.5%), a 40 atoms supercell (containing 15 In atoms, 1 Ti atom, and 24 O atoms) was adopted, and the actual Sn: (In + Sn) ratio was 1:16.

## Conflict of Interest

The authors declare no conflict of interest.

## Author Contributions

W.Y. and Y.W. supervised the project. W.Y. and G.L. conceived the idea and experimental design. X.L. synthesized the material. X.L., M.T.L, Q.Z., and N.L. analyzed and interpreted the experimental data. Y.W. performed density functional theory calculations. S.P. and T.Y. performed TEM measurements. S.G. assisted with the transparency and absorbance measurements. Y.L. and L.Y. assisted with the high‐temperature resistance measurements. J.K. and D.Z. assisted with the synchrotron experiments. X.L. wrote the manuscript. G.L., W.Y. and M.T.L. revised the manuscript. All authors discussed the results and commented on the manuscript.

## Supporting information

Supporting Information

## Data Availability

The data that support the findings of this study are available in the supplementary material of this article.
