# Supplementary material for: Pressure Engineering Promising Transparent Oxides with Large Conductivity Enhancement and Strong Thermal Stability
Source: Adv Sci (Weinh). 2022 Sep 30;9(31):2202973. doi: 10.1002/advs.202202973 (PMC9631087; doi:10.1002/advs.202202973)
Supplement: Supplementary file 1 — Supporting Information [file ADVS-9-2202973-s001.pdf]

Supporting Information

Pressure Engineering Promising Transparent Oxides with Large Conductivity Enhancement and Strong Thermal Stability

Xuqiang Liu, Mingtao Li, Qian Zhang, Yiming Wang, Nana Li, Shang Peng, Tao Yin, Songhao Guo, Ye Liu, Limin Yan, Dongzhou Zhang, Jaeyong Kim, Gang Liu, Yandong Wang*, Wenge Yang**

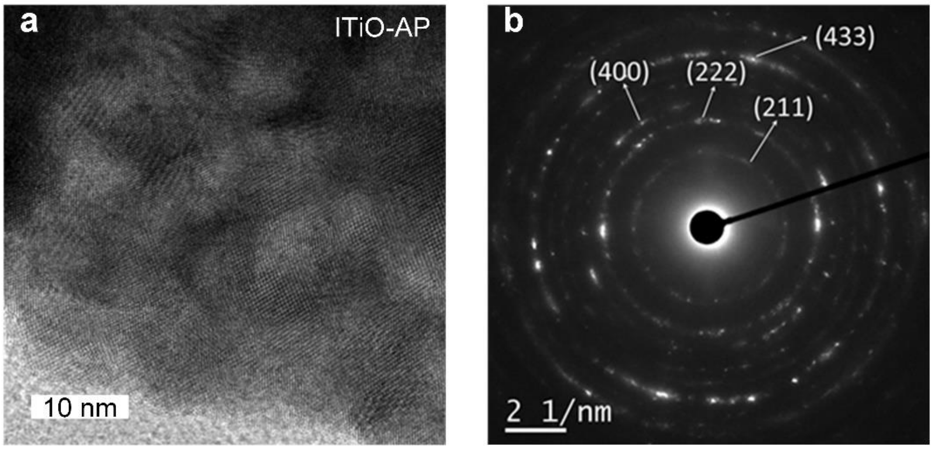

Figure S1. The characterization of Transmission electron microscopy (TEM) on the as-prepared indium titanium oxide (ITiO-AP). a) TEM image of the ITiO-AP at room temperature. b) Selected area electron diffraction (SAED) pattern of the ITiO-AP.

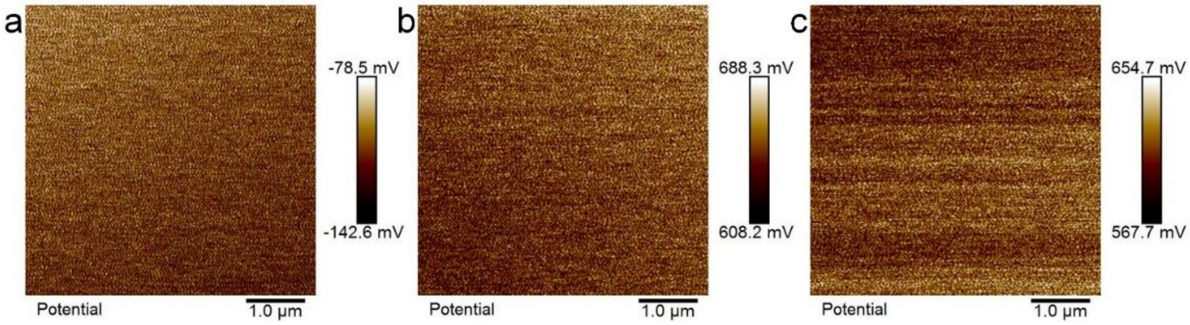

Figure S2. The contact potential difference (CPD) measured by a Kelvin probe. a) The CPD of Au. b) The CPD of ITiO-AP. c) The CPD of ITiO-RP.

Work function is a very important parameter for TCO materials, especially when TCO materials are used as transparent electrode materials in photovoltaic cells. Work function mismatches in photovoltaic cells can lead to undesirable Schottky contacts between the TCO

layer and the semiconductor, resulting in high contact resistance and reduced device efficiency.[1, 2, 3] In a typical thin-film solar cell, the ideal anode prefers a high work function for efficient holes injection, while the ideal cathode prefers a low work function for efficient electrons injection. Work function is highly relevant to the processing method, doping, surface modification, and post-treatment of transparent electrode materials. To understand the effect of pressure treatment (compression-decompression cycle) on the work function properties, we performed Kelvin probes on ITiO-AP and ITiO-RP to study the contact potential difference (CPD), which is directly related to the relative work function. The Kelvin probe results on CPD for Au, ITiO-AP, and ITiO-RP are shown in Figure S2. By comparing them with the CPD of Au, the work functions of ITiO-AP and ITiO-RP are estimated to be 4.38 V and 4.34 V, respectively, which is significantly lower than that of conventional vacuum-deposited ITO (~4.7 V),[4] and ITiO-RP has slightly improved the work functions.

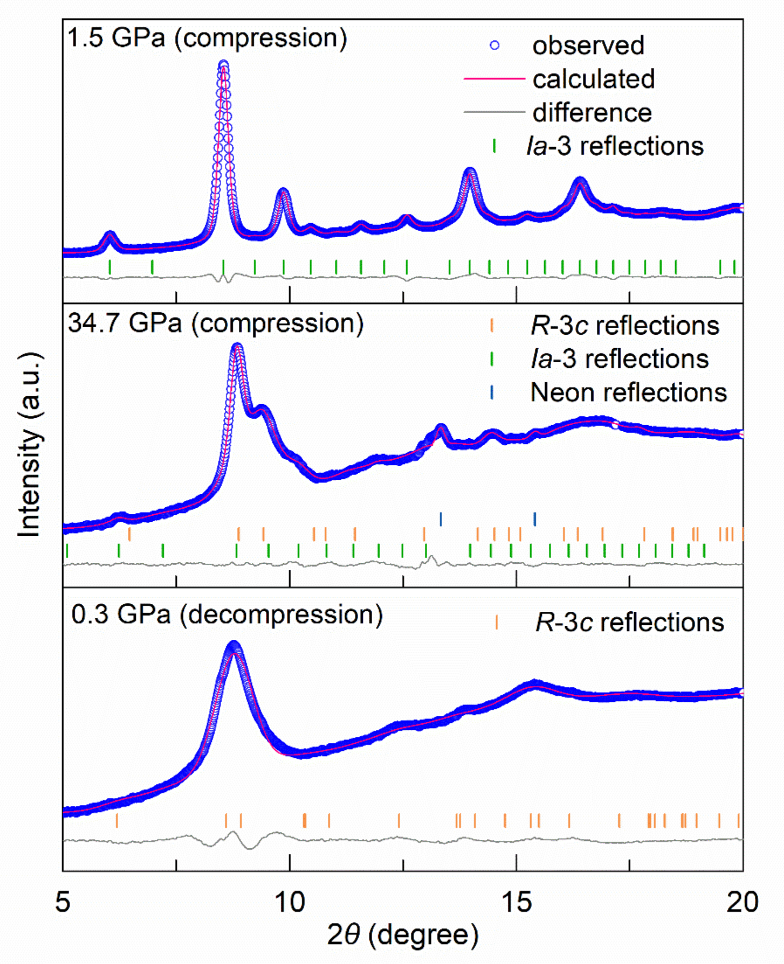

Figure S3. Typical Rietveld refinements of indium titanium oxide X-ray diffraction (XRD) patterns collected during compression-decompression at room temperature.

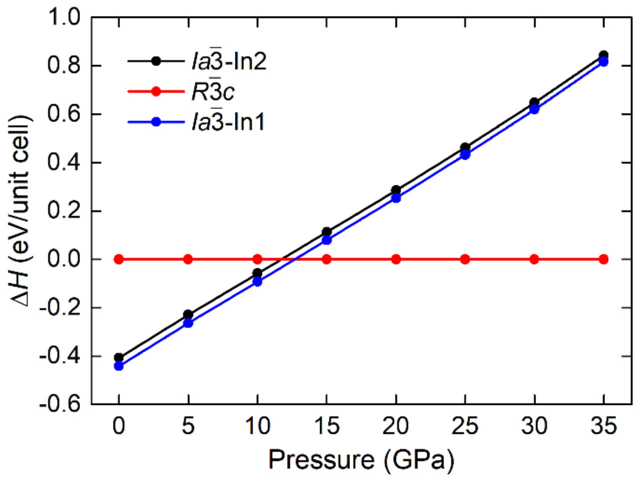

Figure S4. Calculated enthalpies of the $Ia\bar{3}$ phases (relative to the $R\bar{3}c$ phase) for ITiO as a function of pressure.

The summary of literature on compression behavior of In_2O_3

Previous literatures[5, 6, 7, 8, 9, 10] reported controversial results about the high-pressure phase transition behavior of In_2O_3 materials, indicating that In_2O_3 compression behavior remains mysterious. A phase transition of $Ia\bar{3}\rightarrow R\bar{3}c$ has been observed in bulk In_2O_3 and 6 nm nanoparticles In_2O_3.[5, 6] In contrast, no sign of a phase transition was observed in either cubic In_2O_3 bulk material or 9 nm nanocrystals at pressures up to 30 GPa.[7] Furthermore, a complicated phase transition sequence ($Ia\bar{3}\rightarrow Pbcn\rightarrow Pbca\rightarrow R\bar{3}c$) was observed in the compression-decompression cycle of In_2O_3.[8] During colloidal In_2O_3 nanocrystals synthesis, the $R\bar{3}c$ phase may become more stable than the $Ia\bar{3}$ when the In_2O_3 particle size is reduced to below 5 nm.[9] The surface stress seems to play a role in the structural stability of the high-pressure $R\bar{3}c$ phase. High-pressure polymorphs can be stabilized when the particle size is reduced enough to generate surface stress higher than the pressure required for the phase transition.[10] However, it is well known that the starting material's size, defect, stress, and doping elements may affect the structural sequence under pressure. Therefore, an in-depth study into these effects on the compression behavior of In_2O_3 or doped In_2O_3 is still needed to resolve the conflicting results from existing studies.

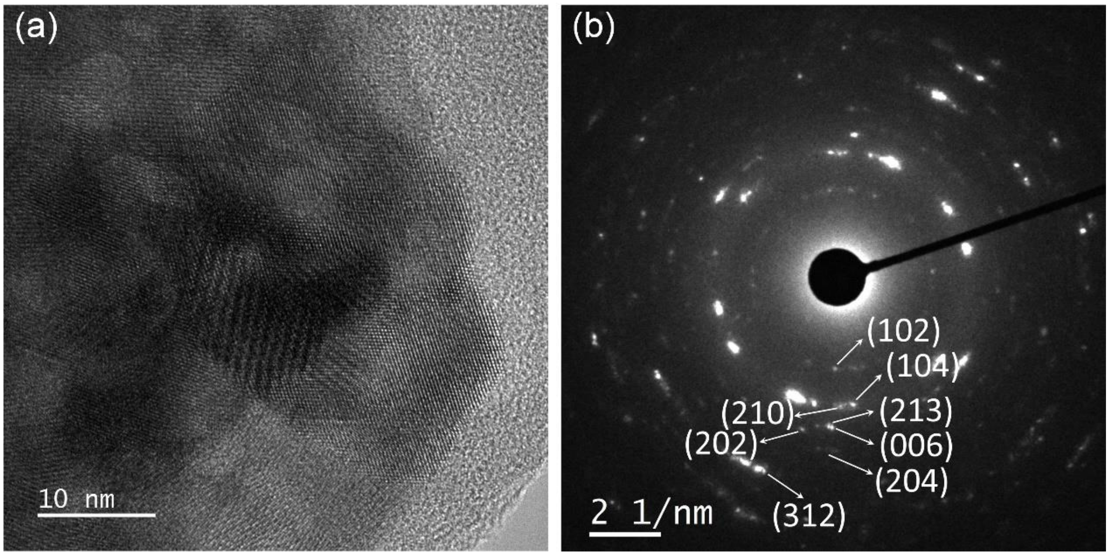

Figure S5. TEM results of the sample after pressure treatment (ITiO-RP). a) HRTEM of the decompressed material. b) SAED pattern of the decompressed material.

Table S1. Comparison of *d*-spacing between XRD-GSAS and TEM-SAED (ITiO-RP).

hkl	*d*-spacing [Å] (XRD-GSAS)	*d*-spacing [Å] (TEM-SAED)
{102}	4.014	4.003
{104}	2.891	2.867
{210}	2.788	2.827
{213}	2.412	2.453
{006}	2.406	2.423
{202}	2.290	2.321
{204}	2.007	2.105
{312}	1.769	1.735

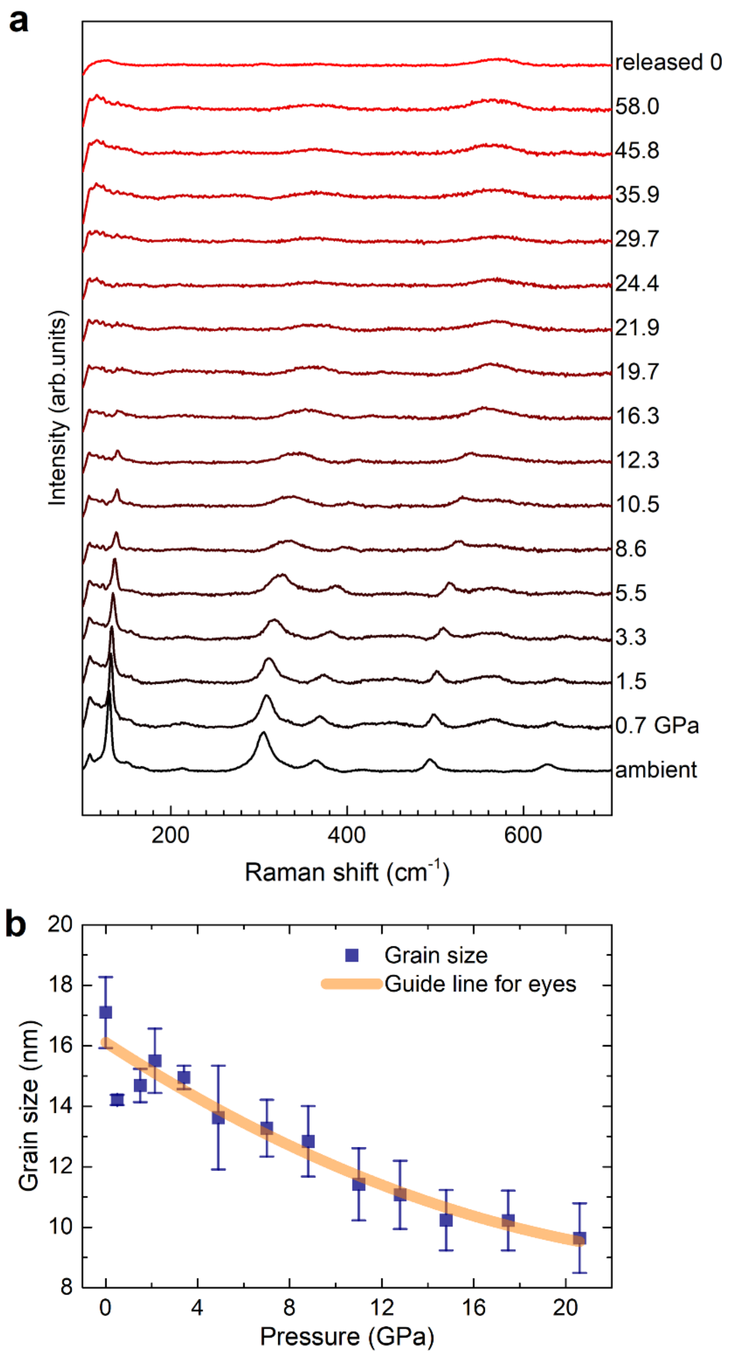

Figure S6. a) Raman-scattering spectra of ITiO at different pressures up to 58.0 GPa at room temperature. b) The grain size of ITiO (the $Ia\bar{3}$ phase) during compression.

High-pressure Raman-scattering experiments were conducted to study the response of vibrational properties to pressure. The Raman spectra of ITiO under compression and decompression are shown in Figure S6a. At ambient pressure, the observed Raman modes are in good agreement with a report on pure In_2O_3.[7] Under compression, we noted that the Raman peaks gradually broadened and their intensity continuously decreased, which exhibits the grain size effect on the Raman spectrum described in previous reports on metal oxides.[11,

[12, 13, 14] By line width analysis from the X-ray diffraction patterns,[15] the grain size of our sample as a function of pressure is plotted in Figure S6b, which shows a reducing trend with compression, supporting the grain size effect on the Raman spectrum. After 16.3 GPa, all Raman modes almost disappear, which is possibly related to structural distortion after the phase transition.[5] Only several peaks with very severe broadening remained in the Raman spectrum until 58 GPa. After releasing pressure, the Raman spectrum stays unchanged compared to that at the highest pressure, verifying the irreversibility of the phase transition.

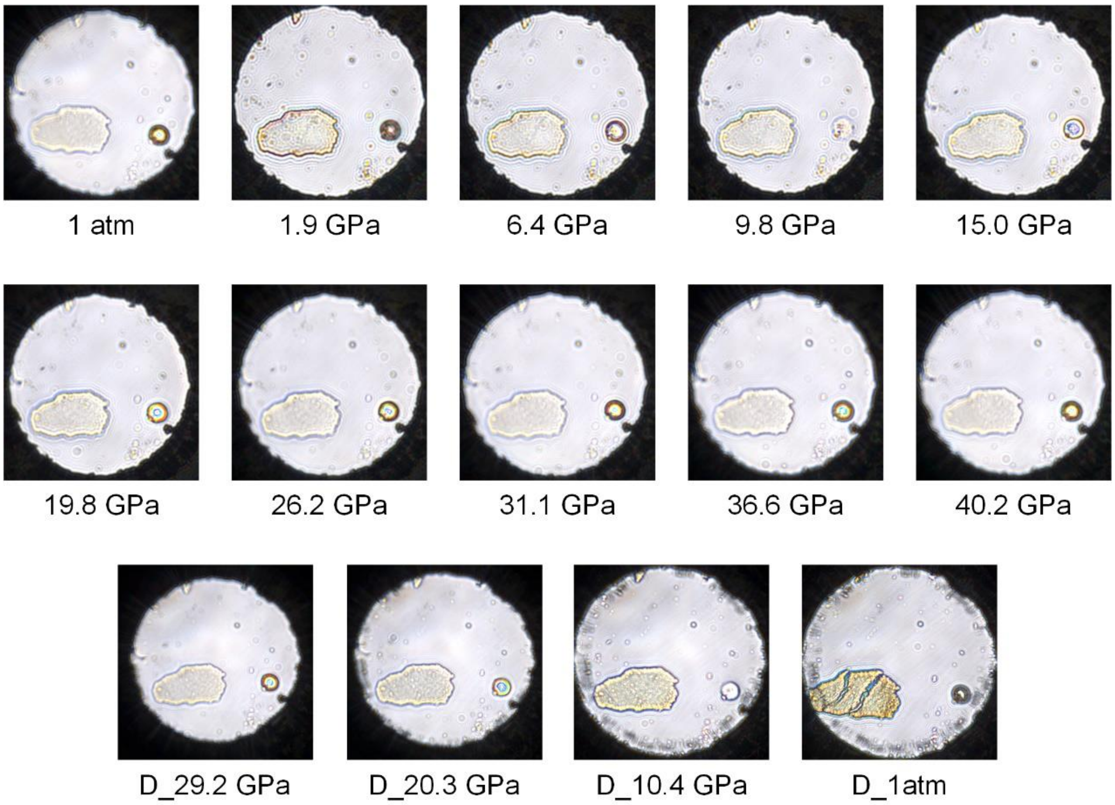

Figure S7. The optical images of ITiO under a compression and decompression cycle at room temperature.

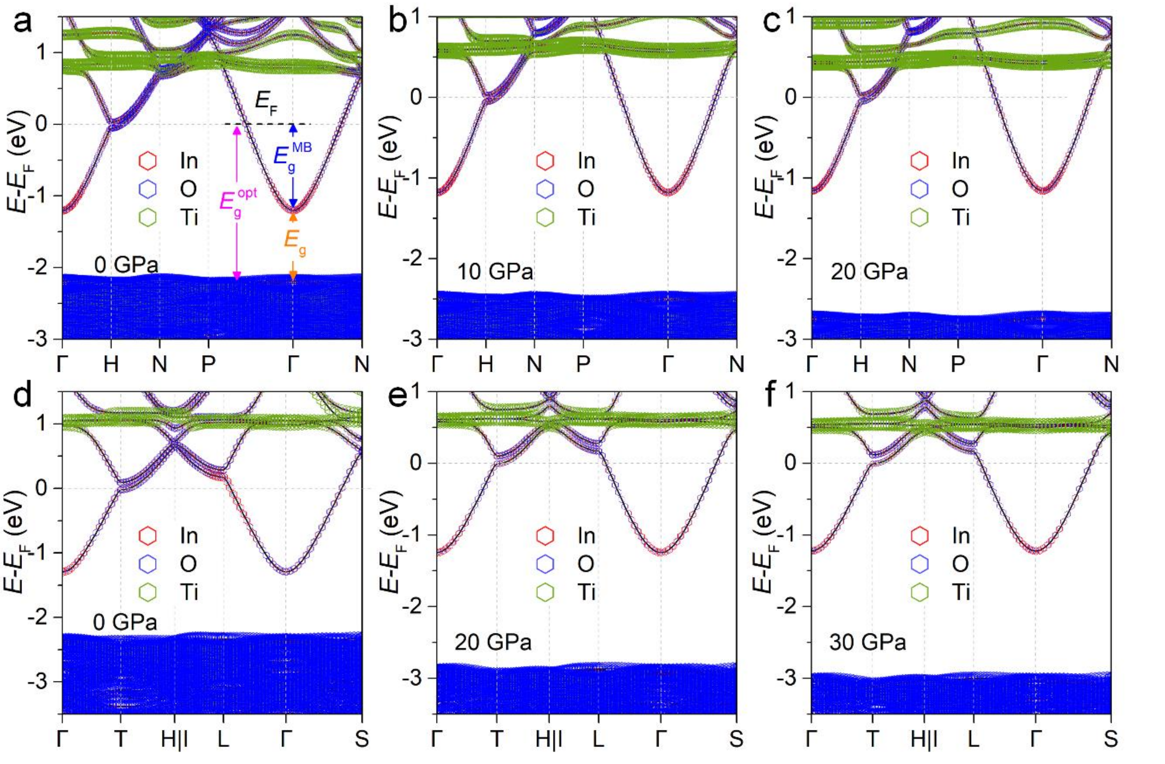

Figure S8. Calculated band structure for two phases of ITiO**.** a-c) The band structure of the $Ia\bar{3}$ phase at 0, 10, and 20 GPa, respectively. d-f) The band structure of the $R\bar{3}c$ phase at 0, 20, and 30 GPa, respectively.

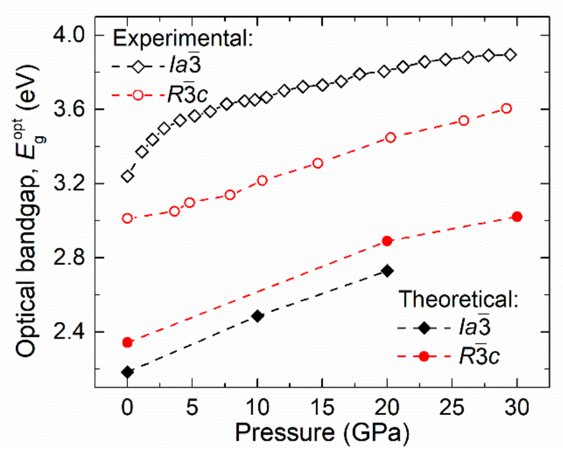

Figure S9. The comparison between the calculated and observed optical bandgap E_g^{opt} of the $Ia\bar{3}$ and $R\bar{3}c$ phases at different pressures.

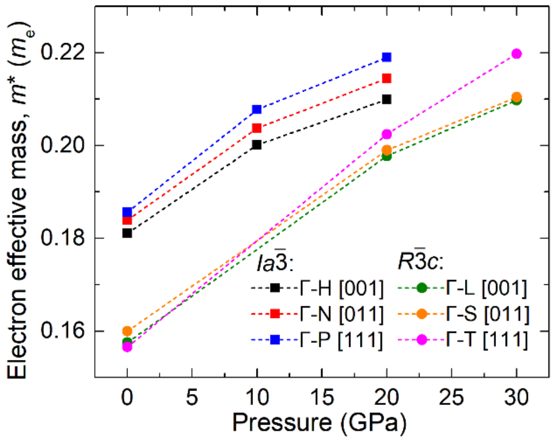

Figure S10. Calculated effective mass for the conduction band electrons of two phases in ITiO under pressure. Here, the second-order derivative calculated by polynomial fitting is used in the effective mass (m^*) calculation with the following equation: $\frac{\partial^2 E(\mathrm{k})}{\partial \mathrm{k}^2} = \frac{\hbar^2}{m^*}$. Note that the effective masses along with the crystallographic directions [001], [011], and [111] correspond to the directions $\Gamma \rightarrow H$, $\Gamma \rightarrow N$, and $\Gamma \rightarrow P$, for the $Ia\bar{3}$ phase and $\Gamma \rightarrow L$, $\Gamma \rightarrow S$, and $\Gamma \rightarrow T$ for the $R\bar{3}c$ phase, respectively.

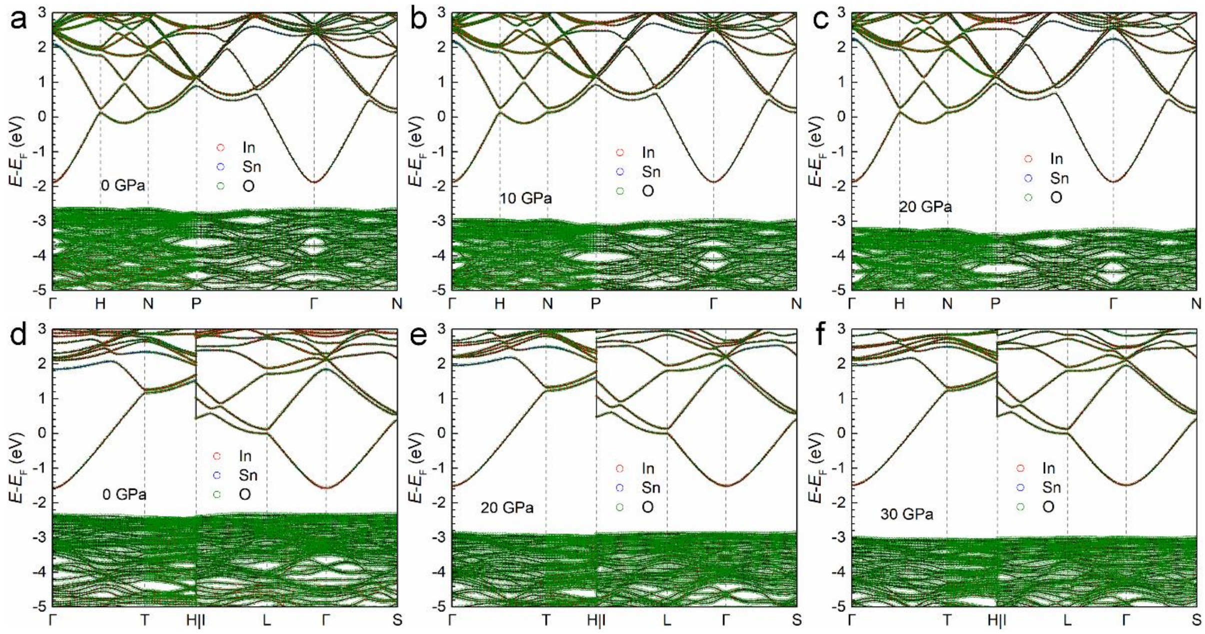

Figure S11. Calculated band structure for two phases of ITO. a-c) The band structure of $Ia\bar{3}$ phase at 0, 10, and 20 GPa, respectively. d-f) The band structure of $R\bar{3}c$ phase at 0, 20, and 30 GPa, respectively.

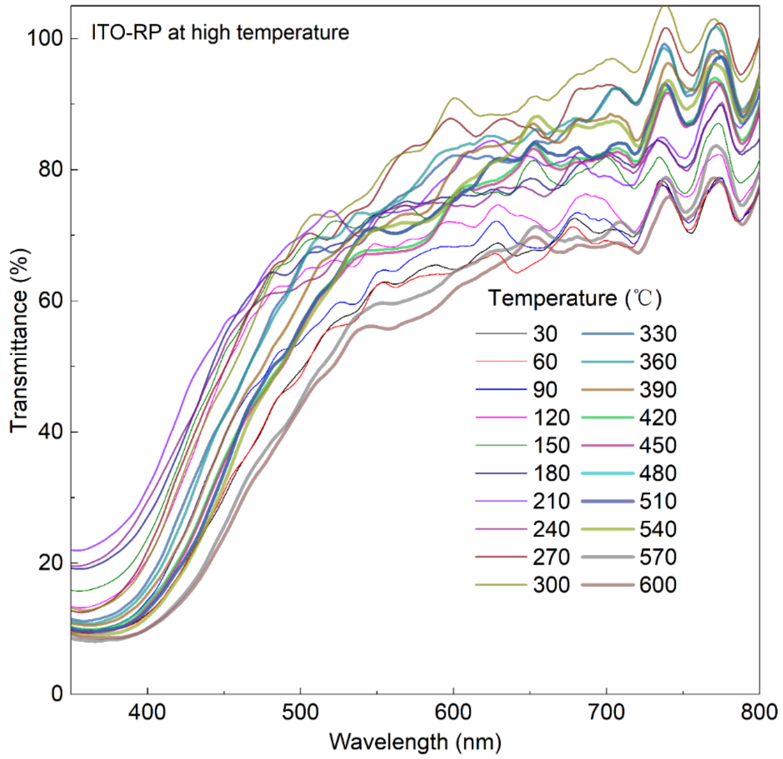

Figure S12. The transmittance spectra of indium titanium oxide after pressure treatment (ITiO-RP) at different temperatures.

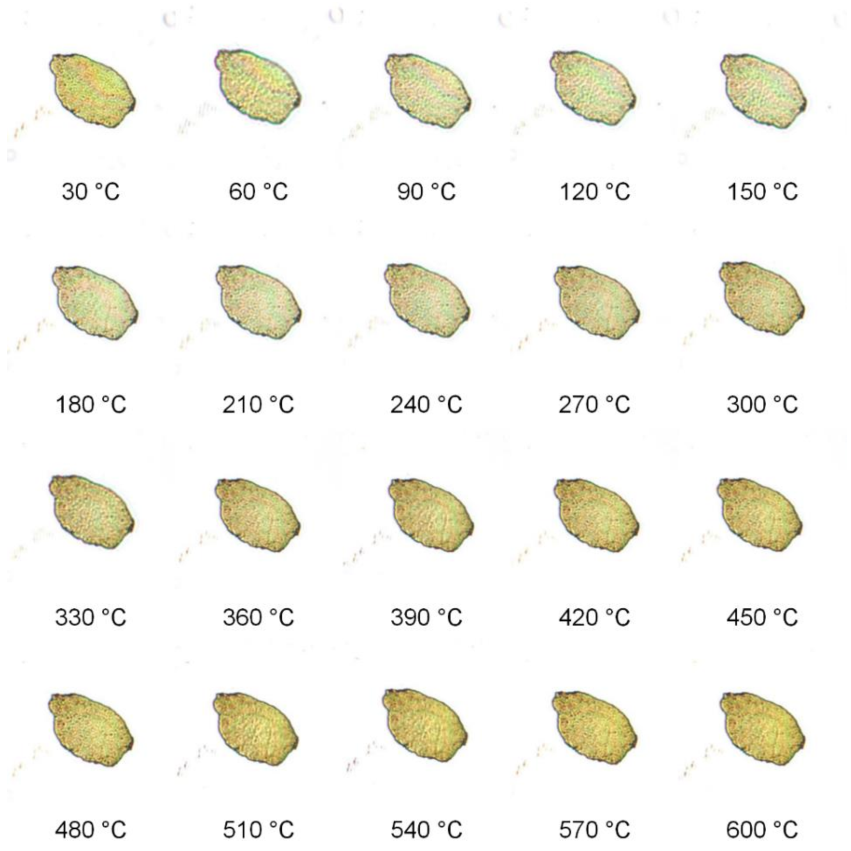

Figure S13. The optical images of ITiO-RP at different temperatures.

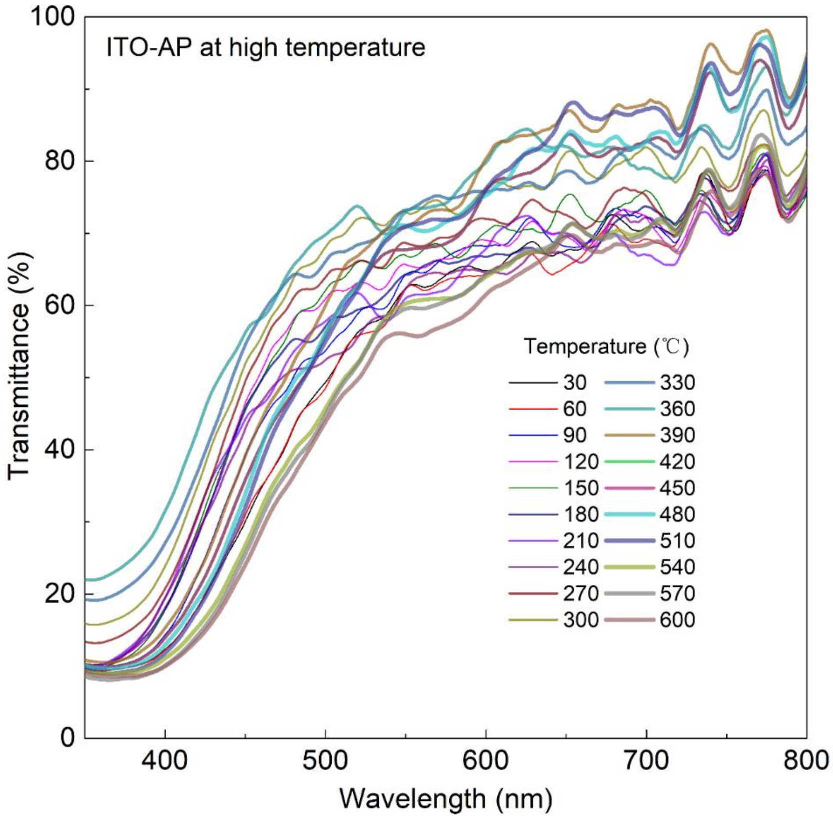

Figure S14. The transmittance spectra of ITiO-AP at different temperatures

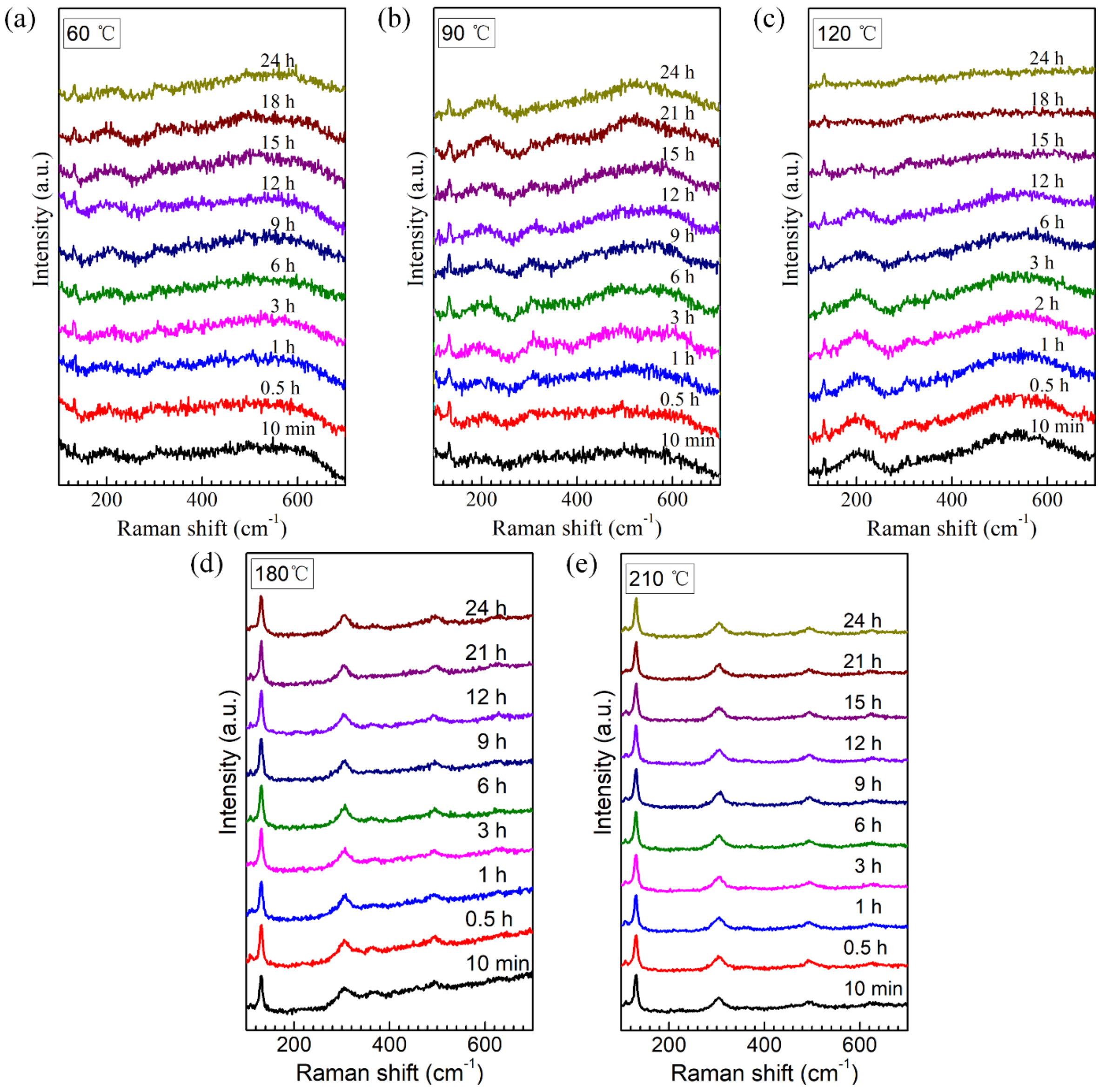

Figure S15. Raman spectrum of ITiO-RP at different temperatures and heating times. a-e) Raman spectrum at 60 °C, 90, 120, 180, and 210 °C.

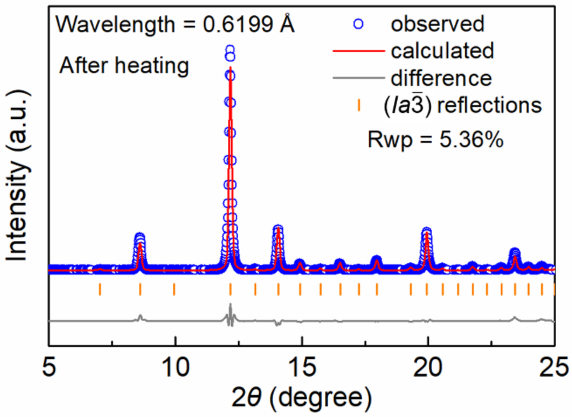

Figure S16. XRD pattern and Rietveld refinement of ITiO-RP after heat treatment at room temperature.

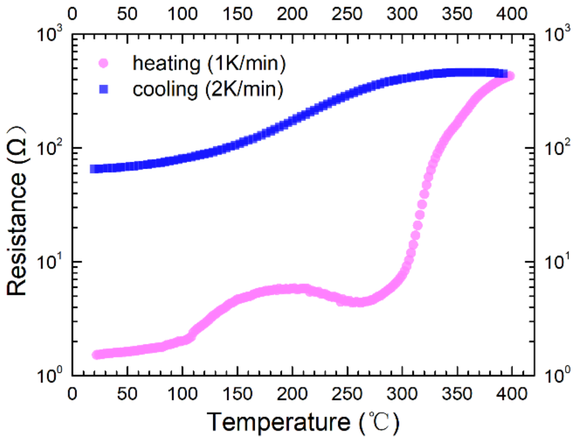

Figure S17. The ITiO-RP resistance during heating treatment.

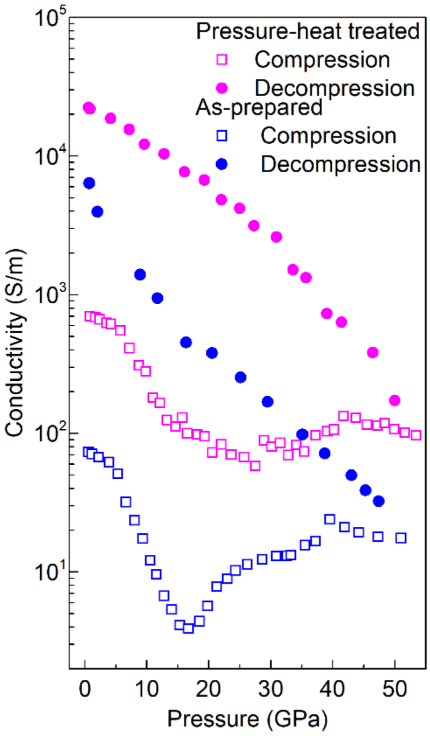

Figure S18. A comparison of the pressure-dependent conductivity between the pressure-heat treated sample and as-prepared sample.

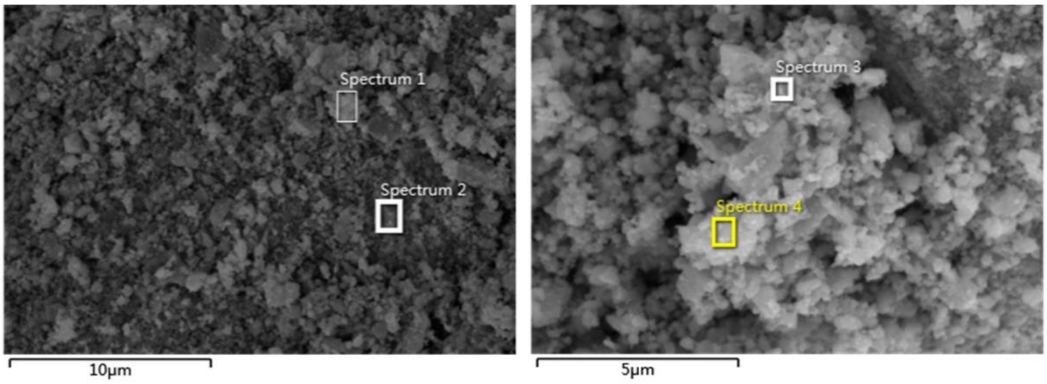

Figure S19. The scanning electron microscopy images of the pristine ITiO-AP sample. Energy dispersion X-ray spectra (EDS) were collected at the four locations marked, and the compositions are summarized in Table S2.

Table S2. EDS results of Ti-doped In_2O_3 nanocrystals

Element	Ti [Atomic %]	In [Atomic %]	O [Atomic %]	Ti/(Ti+In) [%]
Spectrum1	1.77	64.98	33.26	2.65
Spectrum2	1.90	76.53	21.57	2.42
Spectrum3	1.73	61.03	37.25	2.76
Spectrum4	1.65	64.41	33.94	2.50

References

[1] Z. He, C. Zhong, S. Su, M. Xu, H. Wu, Y. Cao, *Nat. Photonics* **2012**, *6*, 591.
[2] M. Morales-Masis, S. De Wolf, R. Woods-Robinson, J. W. Ager, C. Ballif, *Adv. Electron. Mater.* **2017**, *3*, 1600529.
[3] D. Wöhrle, D. Meissner, *Adv. Mater.* **1991**, *3*, 129.
[4] R. Sheats James, H. Antoniadis, M. Hueschen, W. Leonard, J. Miller, R. Moon, D. Roitman, A. Stocking, *Science* **1996**, *273*, 884.
[5] D. Liu, W. W. Lei, B. Zou, S. D. Yu, J. Hao, K. Wang, B. B. Liu, Q. L. Cui, G. T. Zou, *J. Appl. Phys.* **2008**, *104*, 083506.
[6] J. Qi, J. F. Liu, Y. He, W. Chen, C. Wang, *J. Appl. Phys.* **2011**, *109*, 063520.
[7] B. Garcia-Domene, H. M. Ortiz, O. Gomis, J. A. Sans, F. J. Manjón, A. Muñoz, P. Rodríguez-Hernández, S. N. Achary, D. Errandonea, D. Martínez-García, A. H. Romero, A. Singhal, A. K. Tyagi, *J. Appl. Phys.* **2012**, *112*, 123511.
[8] B. García-Domene, J. A. Sans, O. Gomis, F. J. Manjón, H. M. Ortiz, D. Errandonea, D. Santamaría-Pérez, D. Martínez-García, R. Vilaplana, A. L. J. Pereira, A. Morales-García, P. Rodríguez-Hernández, A. Muñoz, C. Popescu, A. Segura, *J. Phys. Chem. C* **2014**, *118*, 20545.
[9] S. S. Farvid, N. Dave, P. V. Radovanovic, *Chem. Mater.* **2010**, *22*, 9.
[10] A. Gurlo, *Angew. Chem., Int. Ed. Engl.* **2010**, *49*, 5610.
[11] J. Zuo, C. Xu, Y. Liu, Y. Qian, *Nanostruct. Mater.* **1998**, *10*, 1331.
[12] G. G. Siu, M. J. Stokes, Y. Liu, *Phys. Rev. B* **1999**, *59*, 3173.
[13] D. Shuo, L. Jin-Quan, L. Yu-Long, *Chin. Phys.* **2004**, *13*, 1854.
[14] G. Gouadec, P. Colomban, *Prog. Cryst. Growth Charact. Mater.* **2007**, *53*, 1.
[15] A. K. Singh, A. Jain, H. P. Liermann, S. K. Saxena, *J. Phys. Chem. Solids* **2006**, *67*, 2197.
